# High Occurrence of a Missense Variant (c.471C>A) in the *FGF23* Gene Related to Hyperostosis–Hyperphosphatemia Syndrome With a Possible Founder Effect

**DOI:** 10.1155/humu/6382674

**Published:** 2025-02-18

**Authors:** Maryam Sedghi, Elika Esmaeilzadeh Gharehdaghi, Vahid Ziaee, Farzaneh Abbasi, Hamid Reza Aghaei Meybodi, Elina Smiley, Mehrzad Mehdizadeh, Seyyed Reza Raeeskarami, Nahid Aslani, Sahar Naderi Shiran, Mehdi Vafadar, Mahsa M. Amoli

**Affiliations:** ^1^Metabolic Disorders Research Center, Endocrinology and Metabolism Molecular-Cellular Sciences Institute, Tehran University of Medical Science, Tehran, Iran; ^2^Department of Pediatrics, Tehran University of Medical Sciences, Tehran, Iran; ^3^Pediatric Rheumatology Research Group, Rheumatology Research Center, Tehran University of Medical Sciences, Tehran, Iran; ^4^Growth and Development Research Center, Children's Medical Center Hospital, Tehran University of Medical Sciences, Tehran, Iran; ^5^Evidence Based Medicine Research Center, Endocrinology and Metabolism Clinical Sciences Institute, Tehran University of Medical Sciences, Tehran, Iran; ^6^CLS Department, School of Health Profession, The University of Texas Medical Branch, Galveston, Texas, USA; ^7^Department of Radiology, Tehran University of Medical Sciences, Tehran, Iran; ^8^Department of Pediatric, Ali Asghar Children's Hospital, School of Medicine, Iran University of Medical Sciences, Tehran, Iran

**Keywords:** *FGF23*, HSS syndrome, mutation c.471C>A

## Abstract

**Background:** The autosomal recessive metabolic disorder hyperostosis–hyperphosphatemia syndrome (HHS) is characterized by hyperphosphatemia, hyperostosis, and recurrent bone lesions. Patients may develop ectopic and vascular calcification and may present diaphyseal pain of the long bones that is misdiagnosed as osteomyelitis. Mutations in *GALNT3* and *FGF23* genes were detected in patients with HHS. The main manifestations of these patients are increased levels of phosphate reabsorption from kidneys and painful swelling of long bones alongside with normal levels of vitamin D and parathormone.

**Method:** We performed whole-exome sequencing (WES) in seven Iranian patients. These patients were referred from several specialist clinics. Seven irrelevant families were examined for genetic mutations.

**Results:** WES revealed the deleterious missense mutation c.471C>A, p. F157L in all affected members of six families. The variant c.1524+1G>A in the *GALNT3* gene was found in the remaining patient which is reported previously. These variants were confirmed utilizing segregation studies in the pedigrees.

**Conclusion:** Our data together with previous studies related to mutations in *FGF23* in Iran strongly support that p. F157L mutation is abundantly prevalent in patients with Iranian origin and is likely to be a founder mutation; however, it requires further confirmative study. The result of this study suggests that p. F157L mutation should be investigated at the first step in genetic analysis of patients with HHS. This enables fast and accurate focused molecular diagnosis and would be effective for use in carrier screening as well as in prenatal diagnosis (PND) and preimplantation genetic diagnosis (PGD).

## 1. Introduction

The autosomal recessive (AR) metabolic disorder hyperostosis–hyperphosphatemia syndrome (HHS) is a rare disorder manifesting with hyperphosphatemia, hyperostosis, and recurrent bone lesions [1]. The main manifestations of these patients are increased levels of phosphate reabsorption from kidneys and recurrent episodes of painful inflammation in long bones alongside with normal levels of vitamin D and parathormone [2]. Mutations in *GALNT3* and *FGF23* genes were detected in patients with HHS [3]. Another AR metabolic disorder is familial hyperphosphatemic tumoral calcinosis (FHTC) which has similarities to HSS in terms of biochemical abnormalities but is different in musculoskeletal pattern [4]. The main characterization of FHTC is ectopic calcification especially in periarticular soft tissues. In addition to loss of function mutations in *FGF23* and *GALNT3* genes, mutations in the *KL* gene encoding alpha-Klotho lead to FHTC [5]. The *FGF23* gene encoding phosphaturic hormone FGF23 is the main regulator of phosphate balance, predominately produced by osteocytes/osteoblasts. FGF23 in proximal renal tubules works through bindings to fibroblast growth factor receptor 1 (FGFR1) and coreceptor Klotho, causing a decline in phosphate reabsorption and inhibit 1,25(OH)_2_D synthesis [6]. Additionally, FGF23 results in inhibiting phosphate absorption from intestines [7]. The *GALNT3* gene encodes UDP-Nacetyl-*α*-D-galactosamine: polypeptide N-acetylgalactosaminyltransferase 3 (GalNAc-T3). This enzyme has a main role in O-linked glycosylation of ^178^Thr in FGF23 and through this action prevents processing of FGF23 between ^179^Arg and ^180^Ser [8]. Inactivation of *GALNT3* due to loss of function mutations impairs the glycosylation and consequently formation of stable FGF23 [9].

In this study, we have presented clinical and genetic findings of seven Iranian FHTC/HHS patients from seven unrelated families. Whole-exome sequencing (WES) revealed the deleterious missense mutation c.471C>A, p. F157L of the *FGF23* gene in all affected members of six families. This mutation was previously reported in three Iranian patients with HHS [10, 11]. Our data together with previous studies related to mutations in *FGF23* in Iran strongly support that p. F157L mutation is abundantly prevalent in HSS patients with Iranian origin and should be considered a founder mutation.

## 2. Material and Methods

### 2.1. Editorial Policies and Ethical Considerations

This study was approved by the Ethical Committee of the Endocrinology and Metabolism Research Institute (ethical code: IR.TUMS.EMRI.REC.1401.051). The consent form was signed by the patient's parents.

### 2.2. Subject and Clinical Investigations

Seven families were included in this study. All the patients had hyperphosphatemia and painful swelling of their long bones. After appropriate genetic counseling, physical and clinical examinations were performed for the patients. All patients had Iranian origin and were originally from southern provinces of Iran.

### 2.3. DNA Extraction, WES, and Bioinformatics Analysis

Blood samples from patients and their parents were collected in EDTA tubes. DNA was extracted from peripheral blood leukocytes by the standard salting-out method [12]. WES was done on the Illumina NovaSeq6000 platform with 100× depth of coverage and 150-bp paired-end reads. Raw sequence data analysis, including base calling, alignment to the hg19 human reference genome (Genome Reference Consortium GRCh37), and variant calling, was performed. We utilized the UnifiedGenotyper tool from GATK tools in the Galaxy online database (http://www.usegaSequencingProject (ESP) and http://evs.gs.washington.edu/EVS) and the Exome Aggregation Consortium (ExAC) database for the annotation level. Filtration of the annotated variants was performed according to their frequency, inheritance pattern, genomic location, type, and function, respectively. We filter out heterozygous variants due to inheritance pattern of HSS in the patient's file. At the end, relevant variants according to the patients' phenotype were found using Phenolyzer [13] and VarElect tools [14]. Pathogenicity of the detected variants was checked by MutationTaster (http://www.mutationtaster.org/), VARSOME (https://varsome.com/), and Franklin (https://franklin.genoox.com/).

### 2.4. Sanger Sequencing Confirmation

Bidirectional Sanger sequencing was performed in the patients and their parents for validation of the identified variants in the WES. Exon 3 of the *FGF3* gene was amplified using specific primers 5⁣′-TAGATGAACTTGGCGAAGGGG-3⁣′ (forward) and 5⁣′-CAGCACTATTTCGACCCGGA-3⁣′ (reverse).

## 3. Results

### 3.1. Clinical and Laboratory Findings

#### 3.1.1. Family 1, IV-1

**Figure 1:**
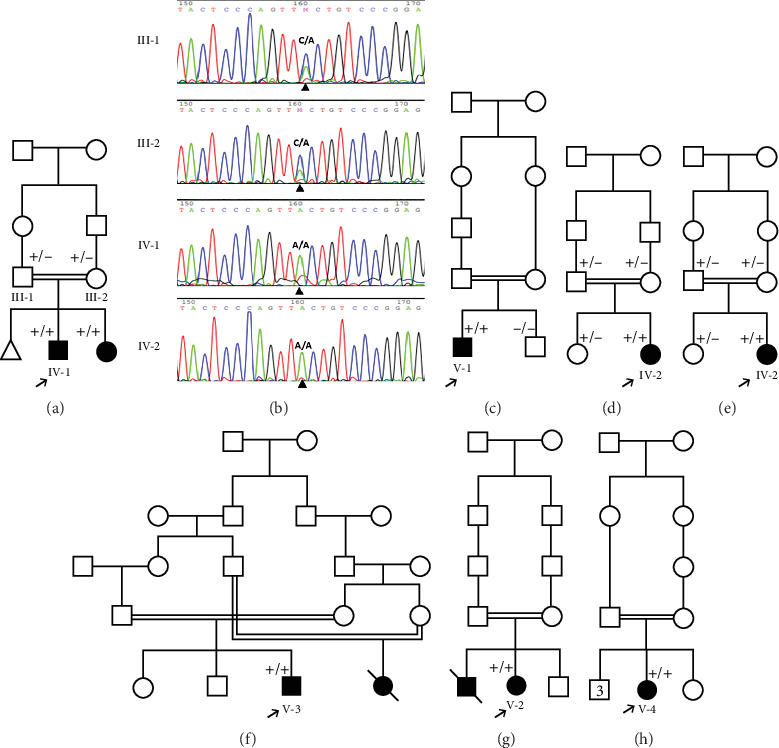
Pedigrees of Families 1–7 with HHS are shown. Shaded symbols −/−, +/−, and +/+ represent wild-type, heterozygous, and homozygous states of the variant c.471C>A, respectively. Sequencing chromatograms in Family 1 are depicted for the patient, his parents, and his affected brother in (b).

This case was a 2-year, 4-month-old boy admitted for evaluation and management of hyperphosphatemia (11.1 mg/dL; normal: 2.7–4.5 mg/dL). Additional serum biochemistries revealed normal calcium (9.2 mg/dL, normal: 8.5–10.4 mg/dL) and 25(OH) vitamin D (20 pg/mL, normal: 15–60 pg/mL) concentrations. He had been experiencing a cough, high fever, coryza, and tearing 40 days before his first visit. He had history of three recurrent facial nerve palsies, and ophthalmological examination revealed bilateral atrophic optic disks and increased latency on visual evoked potential (VEP). Skull CT disclosed hyperostosis of bilateral optic canals that cause obstruction. No additional skeletal disease was detected in physical examination. He was referred to a neurologist, and the brain MRI was normal. He did not respond to high-dose corticosteroids, and his sister (IV-2) was a 3-year, 6-month-old girl who had the same clinical features of bilateral atrophic optic disks, hyperostosis of bilateral optic canals, and facial nerve palsies with no response to high-dose corticosteroids (Figure 1(a)).

The parents of this case were first cousin.

#### 3.1.2. Family 2, V-1

A 7-year-old boy born of a consanguineous marriage (Figure 1(c)) was referred for evaluation and management of fatigue, myalgia, bruising of legs, and hyperphosphatemia (8.3 mg/dL; normal: 2.7–4.5 mg/dL). Additional serum biochemistries revealed normal calcium (9.6 mg/dL, normal: 8.5–10.4 mg/dL) and 25(OH) vitamin D (21 pg/mL, normal: 15–60 pg/mL) concentrations. He had history of facial palsy once at the age of 2 and another at 6 years old. Ophthalmological examination was normal. A skull radiograph revealed hyperostosis and thickening of bilateral optic canals, which remained open. Brain MRI was normal. Sonography of the kidney showed normal-sized kidneys with nephrocalcinosis (the largest one is 9 mm). Audiometry was normal, and no additional skeletal disease was detected in physical examination.

#### 3.1.3. Family 3, IV-2

**Figure 2:**
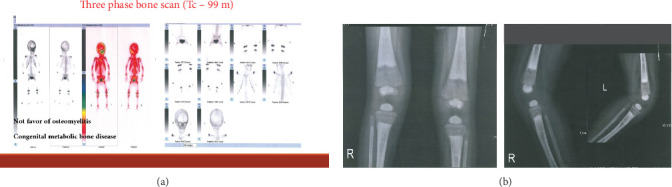
(a) Three-phase bone scan suggesting congenital metabolic bone disease. (b) Knee graph of Patient IV-2 in Family 3.

A 22-month-old girl was referred for evaluation of hyperphosphatemia (10.5 mg/dL; normal: 2.7–4.5 mg/dL). Additional serum biochemistries revealed normal calcium (10 mg/dL, normal: 8.5–10.4 mg/dL) and 25(OH) vitamin D (17 pg/mL, normal: 15–60 pg/mL) concentrations. The second child from consanguine parents and her 5-year-old sister were healthy (Figure 1(d)). She suffered from knee claudication and inability to walk and also fever 1 month before visit and was evaluated in an orthopedic clinic for a probable diagnosis of osteomyelitis, and a three-phase bone scan was performed ruling out osteomyelitis and suggesting congenital metabolic bone disease (Figure 2(a)). She had history of Bell's palsy 7 months before visit that was treated with corticosteroid and discontinued after 40 days. Examination revealed swelling of both knees without effusion and hotness and erythema, and range of motion (ROM) was normal. Radiographs of the knees showed bilateral symmetrical metaphyseal bands (Figure 2(b)). Bilateral hip and knee sonography and abdominal sonography were normal.

#### 3.1.4. Family 4, IV-2

The patient was a 7-year-old girl who was a product of consanguineous marriage (Figure 1(e)) and presented at the age of 4 with poor weight gain, anorexia, and knee pain. According to her parents, she suffered from bilateral knee pain and cried at the slightest impact on her knee. She could not walk or run very well. With this symptom, she was seen by several physicians and given vitamin D supplements. Her symptoms and knee pain worsened with the vitamin D supplements. A subsequent laboratory test revealed elevated phosphate levels. On physical examination, the patient was thin and tall. White deposits were seen on the lid margin and peripheral parts of the tympanic membrane. Her neurologic and dental examinations were normal. She had tender knees with mild valgus deformity without erythema or swelling. The history of fracture was negative. According to her medical history, she had multiple renal calcifications ranging from 2.6 to 6.1 mm in both kidneys. There were no vascular calcifications on ultrasound. On audiometry, there was a certain degree of bilateral conductive hearing loss. The first serum phosphate level was checked at the age of 5 and was 9.9 mg/dL. The level of calcium was 9.4 mg/dL. In subsequent laboratory tests, the percent of tubular phosphate reabsorption (TRP) was 98.5% and the tubular maximum phosphate reabsorption (TmP) to glomerular filtration rate (GFR) was 8.96 mg/dL (normal range 2.9–6.5). The urine calcium-to-creatinine ratio was 0.28. Radiographic evidence of increased intramedullary density was seen in the epiphyses of the long bones of the upper and lower extremities and the calcaneus and talus (“bone within bone” appearance). Cortical thickening of the tibial diaphysis was also observed.

#### 3.1.5. Family 5, V-3

This patient was a 3-year, 4-month-old boy from a consanguineous marriage (Figure 1(f)) with hyperphosphatemia, ptosis, and Bell's palsy. When he was nine months old, his symptoms of Bell's palsy started. Brain MRI showed increased thickness of scalp and increased density of bone. Pigmentary changes in the retina were also observed. VEP was normal. On echocardiography, two small echogenic focuses were seen in LV cavity without cardiac effect.

#### 3.1.6. Family 6, V-2

**Figure 3:**
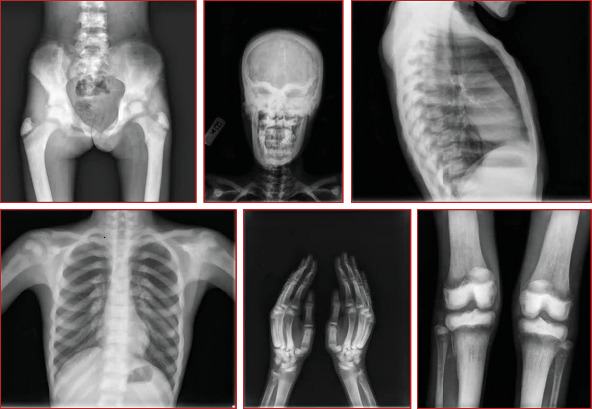
Limb radiographs show increased bone density and increased cortical thickness. Skull X-ray shows lytic lesions and skull base sclerosis.

A 12-year-old female patient was referred to a rheumatology clinic with musculoskeletal problems including claudication and bilateral limping 1 year ago. She also complains of mild bilateral hip pain, generalized weakness, and loss of appetite and weight loss. She had one seizure attack at 7 years old, and she suffered from right facial palsy and shoulder draping at the same side at 10 years old. Her parents were consanguine (Figure 1(g)). She also had an older brother who died because of congenital musculoskeletal disease at 13 years old. Examination revealed long limbs with marfanoid features, strabismus, and abnormal tooth morphology. She had a pigeon chest, also known as pectus carinatum. Small inguinal masses around 3–4 cm with solid texture and a soft mass of 1 cm under the left knee were detectable. On joint examination, limitation in abduction and external rotation of bilateral hips was seen. Lab data revealed hyperphosphatemia (8.4 mg/dL; normal: 2.7–4.5 mg/dL). Additional serum biochemistries revealed normal calcium (10.3 mg/dL, normal: 8.5–10.4 mg/dL) and 25(OH) vitamin D (28 pg/mL, normal: 15–60 pg/mL) concentrations. Radiological examination showed increased bone density and increased cortical thickness and medullary sclerosis of long bones with a significant decrease in density in metaphysis which was also present in hand X-rays. A skull X-ray showed lytic lesions and skull base sclerosis (Figure 3). Results of brain MRI and EEG and echocardiography were normal.

#### 3.1.7. Family 7, V-4

A 47-year-old female patient from a consanguineous marriage (Figure 1(h)) with past medical history of diabetes mellitus, cardiac atherosclerosis, hypertension, hypothyroidism due to Hashimoto disease, and several surgeries of bone lesions presented with bone pain.

Lab data revealed an HbA1c of 7.6% (normal range: 4–5.6%), Mg of 2 mg/dL (normal range: 1.9–2.5 mg/dL), Ca of 10 mg/dL (normal range: 8.5–10.4 mg/dL), Pho of 6.6 mg/dL (normal range: 2.6–4.5 mg/dL), vitamin D3 of 32 ng/mL (normal range: 20–100 ng/mL), PTH of 9.4 pg/mL (normal range: 11–67 pg/mL), urine Ca of 75 mg/24 h (normal < 250/24 h), and urine Pho of 650 mg/24 h (normal range: 400–1300 mg/24 h).

### 3.2. Genetic Analysis and In Silico Predictions

WES results of Individuals IV-1 (Family 1), V-1 (Family 2), IV-2 (Family 3), IV-2 (Family 4), V-3 (Family 5), and V-2 (Family 6) revealed the variant c.471C>A, p. F157L in Exon 3 of the *FGF23* gene.

The abovementioned variant was validated by PCR and bidirectional Sanger sequencing analysis in all available unaffected family members, which was consistent with a heterozygous or homozygous pattern of the wild-type allele (Figure 1). Sequencing chromatograms of the patient in Family 1 and her affected sister showed homozygous missense mutation c.471C>A, while the parents were heterozygous for the mutation (Figure 1(b)).

**Figure 4:**
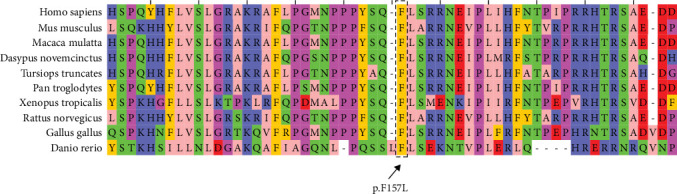
Multiple sequence alignment for amino acids encoded by FGF23 obtained by COBALT (https://www.ncbi.nlm.nih.gov/tools/cobalt/cobalt.cgi) and colored by Jalview. Amino acid phenylalanine 157 is highly conserved among different jawed vertebrates. Zappo color style was used to categorize amino acids based on their physicochemical properties. Amino acids with similar properties have the same color (aliphatic/hydrophobic (I, L, V, A, M) (light pink), aromatic (F, W, Y) (mustard), positively charged (K, R, H) (blue), negatively charged (D, E) (red), hydrophilic (S, T, N, Q) (green), conformationally special (P, G) (magenta), and C (yellow)).

The variant c.471C>A changes the highly conserved amino acid phenylalanine at position 157 to leucine. This mutation is deleterious and probably affecting the heparin binding domain. The sequence of protein FGF23 comprising phenylalanine at position 157 in a set of eight organisms was aligned simultaneously by the COBALT tool and colored by using Jalview. This proposed that phenylalanine 157 is a functional residue and is highly conserved across a large set of species (Figure 4). This variant is pathogenic according to ACMG criteria [15].

The variant c.1524+1G>A; K465_Y508del in the *GALNT3* gene was found after filtration of all identified variants in WES for the proband of Family 7. This variant is a splice site mutation which is located in Intron 8 of the *GALNT3* gene. The variant c.1524+1G>A is a pathogenic variant and causes mRNA missplicing, followed by a nonsense-mediated decay process [16].

## 4. Discussion

**Figure 5:**
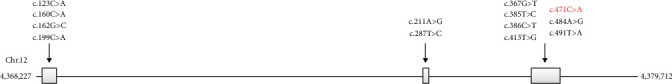
Schematic diagram indicating position of homozygous pathogenic variants with HFTC/HHS.

The *FGF23* gene is located on Chromosome 12 and is comprised of three exons. To date, 13 different mutations in the *FGF23* gene causing FHTC/HHS have been reported in different populations [10, 17–24] (Figure 5). Among them, the variant c.160C>A in Exon 1 of *FGF23* is previously reported in an Iranian pedigree with FHTC [21].

These 13 mutations lead to hyperphosphatemia and increased the absorption of gastrointestinal phosphorus and calcium. Most intragenic mutations are located in Exon 3 resulting in impaired protein glycosylation [6]. O-linked glycosylation in the position of ^178^Thr in FGF23 prevents processing between ^179^Arg and ^180^Ser which is an essential process for the activation of FGF23 [7].

Here, we introduced seven Iranian pedigrees with hyperphosphatemia, hyperostosis, and recurrent bone lesions. Clinical manifestations of all the seven pedigrees in the current investigation were consistent with FHTC/HHS. WES revealed the homozygous variant c.471C>A in Exon 3 of the *FGF23* gene in the six pedigrees who were referred to us from irrelevant families. All the patients were of southern Iranian ethnicity. Previously, this mutation was reported in the three Iranian patients with HSS [11]. Hitherto, the variant c.471C>A has not been reported in other population. In the current investigation, we provided more evidence that the variant c.471C>A is likely displaying a founder effect in the Iranian population with a probable origin in Iran; however, it needs further confirmative study. Although linkage study was not performed, it seems that a common ancestry and the existence of a founder mutation for HHS are likely in the Iranian population. Detection of ethnic-specific mutations would be valuable allowing for more rapid and cost-effective genetic screening. It is not only used for carrier status of a population but also used for facilitating prenatal diagnosis (PND) and preimplantation genetic diagnosis (PGD) for involved pedigrees.

On the other hand, we found the mutation c.1524+1G>A; K465_Y508del in the *GALNT3* gene in the remaining family. This mutation has been previously reported in association with FHTC and HHS in Arab-Moslem and Druze families [16]. The notion is that this mutation has a founder effect in the Middle East. In the current study, we discovered the mutation c.1524+1G>A in a HSS pedigree from southern Iran. This finding can suggest entrance of this variant from the Arab population into southern Iran.

## 5. Conclusion

According to the abovementioned data, we confirmed that the mutation c.471C>A, p.F157L in the *FGF23* gene is a founder mutation in Iranian patients with HSS and should be examined at the first line.

## Data Availability

All data will be available on request.
